# Elements of Histology

**Published:** 1884-09

**Authors:** 


**Elements of Histology.** By E. KLEIN, M. D., F. R. S., Joint Lecturer on General Anatomy and Physiology in the Medical School of St. Bartholomew's Hospital, London.

